# Stratification and prediction of drug synergy based on target functional similarity

**DOI:** 10.1038/s41540-020-0136-x

**Published:** 2020-06-02

**Authors:** Mi Yang, Patricia Jaaks, Jonathan Dry, Mathew Garnett, Michael P. Menden, Julio Saez-Rodriguez

**Affiliations:** 1 Heidelberg University, Faculty of Biosciences, Germany; 2 RWTH Aachen University, Faculty of Medicine, Joint Research Centre for Computational Biomedicine (JRC-COMBINE), Aachen, Germany; 3 Wellcome Trust Sanger Institute, Wellcome Trust Genome Campus, Cambridge, CB10 1SA UK; 4 Oncology, IMED Biotech Unit, AstraZeneca R&D Boston, Waltham, MA USA; 5 Institute of Computational Biology, Helmholtz Zentrum München - German Research Centre for Environmental Health, 85764 Munich, Germany; 6 Department of Biology, Ludwig-Maximilians University Munich, 82152 Martinsried, Germany; 7 German Centre for Diabetes Research (DZD e.V.), 85764 Neuherberg, Germany; 8 Institute for Computational Biomedicine, Heidelberg University Hospital and Heidelberg University, Faculty of Medicine, Bioquant, Heidelberg, Germany; 9 Present Address: Division of Oncology, Department of Medicine, Stanford Cancer Institute, Stanford University, Stanford, CA USA

**Keywords:** Cancer, Pharmacology, Computational biology and bioinformatics

## Abstract

Drug combinations can expand therapeutic options and address cancer’s resistance. However, the combinatorial space is enormous precluding its systematic exploration. Therefore, synergy prediction strategies are essential. We here present an approach to prioritise drug combinations in high-throughput screens and to stratify synergistic responses. At the core of our approach is the observation that the likelihood of synergy increases when targeting proteins with either strong functional similarity or dissimilarity. We estimate the similarity applying a multitask machine learning approach to basal gene expression and response to single drugs. We tested 7 protein target pairs (representing 29 combinations) and predicted their synergies in 33 breast cancer cell lines. In addition, we experimentally validated predicted synergy of the BRAF/insulin receptor combination (Dabrafenib/BMS-754807) in 48 colorectal cancer cell lines. We anticipate that our approaches can be used for prioritization of drug combinations in large scale screenings, and to maximize the efficacy of drugs already known to induce synergy, ultimately enabling patient stratification.

## Introduction

In the quest for clinical efficacy, drug combinations are a promising strategy in cancer treatment^1,2^. Targeting a signaling pathway at one step may not be sufficient for reaching maximal effects on pathway inhibition. Using one agent at higher dose could be a short-term solution. However, higher dose leads to increased toxicity and emergence of resistance to treatments. Resistance mechanisms to monotherapy can occur by activation of compensatory signaling. For example, the activation of ERK signaling in melanoma when treated with BRAF inhibitors may lead to paradoxical activation of CRAF^3^. Targeting BRAF and downstream MEK at the same time proved to be beneficial for overall patient survival^4^, by inhibiting the initial BRAF driver mutation and paradox CRAF activation. Alternatively to inhibiting two key proteins within the same pathway, a common strategy is to inhibit in parallel two separate cancer pathways to maximize drug efficacy. For example, parallel inhibition of ERK and AKT could be beneficial as those pathways may be connected through cross talks and feedback loops in breast cancer^5^. Given the enormous space of potential drug combinations, strategies to effectively predict their efficacy are highly desirable.

Many methods predict drug synergy using chemical structure and genomic information^6–8^. Preuer et al.^8^ used deep learning to predict synergy within the space of explored drugs and cell lines (Pearson’s correlation of observed versus predicted synergy score *r* = 0.73), but observed a much worse performance in predicting untested drugs (*r* = 0.48) or untested cell lines (*r* = 0.57)^8^. Jaeger et al.^9^ identified new drug combinations using network topology of pathway cross-talk^9^. However, gene mutation information, arguably the most actionable information in the clinic, was not used. In the recent dialog on reverse-engineering assessment and methods (DREAM) drug combination challenge^10^, the best performing team used a protein–protein interaction network to augment the genomic features based on their network distance from drug targets. While the best performer achieved outstanding predictability comparable to the level of experimental replicates, synergy was predicted based on supervised machine-learning algorithms. A common bottleneck for the application of all supervised learning methods is the limited publicly available combinatorial drug screening data. In practice, the combinatorial explosion of drug pairs is the limiting factor to both the number of experimentally tested drugs, and the number of tested cell lines. In addition, tested combinations are driven by expert’s knowledge, and therefore may be focused on known biological examples and thereby bias the performance of supervised learning.

Synergy of combination can be estimated from the effect of single drugs. For example, in the NCI-DREAM Drug Sensitivity and Drug Synergy Challenge^7^ the similarities on the effect of drugs on gene expression was used to predict synergy. However, this requires the generation of expression data upon treatment with the drugs, which is relatively costly.

We here investigate if we can use only the similarities of single drugs in their effect on cell survival to learn about the efficacy of combinations. We propose a methodology for prioritizing drug combinations and for cell line stratification based on the functional similarity between two target proteins. For this, we extend the notion of compound similarity to target similarity: the functional similarity of a pair of target proteins is defined as the correlation between the drug response upon perturbation of those proteins, as a function of the activity of a set of essential pathways. Pathway activities are computed from basal gene expression, using data-derived gene sets, that have been demonstrated to be more predictive than pathway-based gene sets^11,12^. Different cancer types may be driven by different cancer pathways. Therefore, the similarity metric is context dependent. Two target proteins that are functionally very similar are likely to belong to the same signaling pathway; on the contrary, functionally dissimilar proteins are likely to belong to unrelated pathways. We find higher synergy likelihood when there is either very high or very low similarity. Based on this information, we build a compound prioritization methodology for high-throughput screens, that does not require any data on the response to any drug combination. Furthermore, we explore context specific (breast and colorectal cancer) drug combinations for their mode of actions based on known mechanistic insights from the monotherapies, to predict synergy and potentially enable patient stratifications in the clinic.

## Results

The fundamental concept underlying our approach is the functional similarity in drug response profile with respect to a set of essential pathways when targeting two proteins. We illustrate its application with the data from the Genomics of Drug Sensitivity in Cancer (GDSC) data^13^ composed of 990 cancer cell lines, treated by 265 drugs, with deep molecular characterization of the cell lines including gene expression, methylation, DNA mutation, and copy number variation profiles. In addition, the nominal target for the drugs is known. For our approach, the basal gene expression profile of the cell lines, their response to the drugs (IC50), and the nominal targets of the drugs are needed.

### Profiling target functional similarity with matrix factorization

**Fig. 1:**
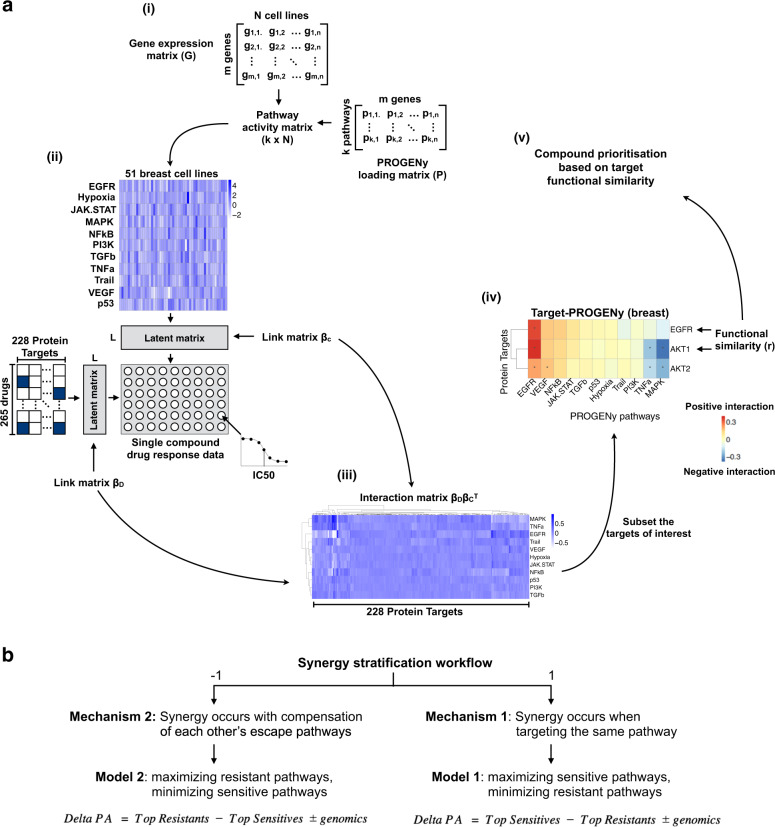
Methodology for drug synergy prediction and stratification. **a** (i) First, we compute activities scores for 11 pathways from gene expression of cancer cell lines. It consists in multiplying the transcriptomics data by a loading matrix using PROGENy^12^. (ii) We then use the Macau algorithm^14^ to predict multiple drugs’ responses simultaneously by uncovering the common (latent) features that can benefit each individual learning task. We use as input features (side information) the PROGENy pathway scores) for the cell lines and the nominal target for the drugs. Each side information matrix is transformed into a matrix of L latent dimensions by a link matrix. Drug response is then computed by a matrix multiplication of the two latent matrices. (iii) Concurrently to drug response prediction, we derive the interactions between drug features (targets) and cell line features (pathway activity), by multiplying the two link matrices. An association between protein X and pathway Y means that activation of pathway Y correlates with drug sensitivity when targeting protein X. In case of causality, we can say that activation of pathway Y confers sensitivity to any drug targeting protein X. The approach behind steps (i–iii) is described in detail in Yang et al.^15^. (iv) These interactions allow us to define the functional similarity between two target proteins. In this example of breast tissue, the functional similarity between proteins EGFR and AKT1 is the correlation of their interaction values with the 11 PROGENy pathways. As the final step of the synergy prediction workflow, the derived target functional similarity informs us about the likelihood of synergy. (v) We use the Target functional similarity metric for compound prioritization. **b** For synergy stratification workflow, we start with target pairs already known to be synergistic. The value of the functional similarity between the target proteins reflects different synergy mechanisms. If the similarity is close to 1, synergy occurs by targeting the same signaling pathways. A similarity close to −1 suggests a synergy induced by compensation of escape mechanism. We build specific synergy models for each case to predict synergy scores of cancer cell lines. Long arrows denote transition to a new step and short arrows indicate direction of matrix multiplications.

We first computed the activity of 11 pathways (EGFR, NFkB, VEGF, JAK-STAT, TGBf, p53, Hypoxia, Trail, PI3K, TNFa, and MAPK) for all cell lines from their basal gene expression, as provided by GDSC, using Pathway RespOnsive GENes (PROGENy^12^, “Methods”, Fig. 1a). Next, we applied the Macau algorithm^14^ to find interactions between the drugs’ nominal targets and pathway activities, as described in Yang et al.^15^ (“Methods”, Fig. 1a). We considered two target proteins, each targeted by a different drug, and took the Pearson’s correlation between their interactions with all PROGENy pathways. We defined this correlation as the functional similarity of those two proteins. A pathway contains more information than a single gene’s expression level. Therefore, functional similarity based on a small subset of essential pathways is likely to be more robust than using thousands of genes, of which the vast majority are not involved in drug response or in cancer.

### Functional similarity of drugs’ targets influences drug synergy

**Fig. 2:**
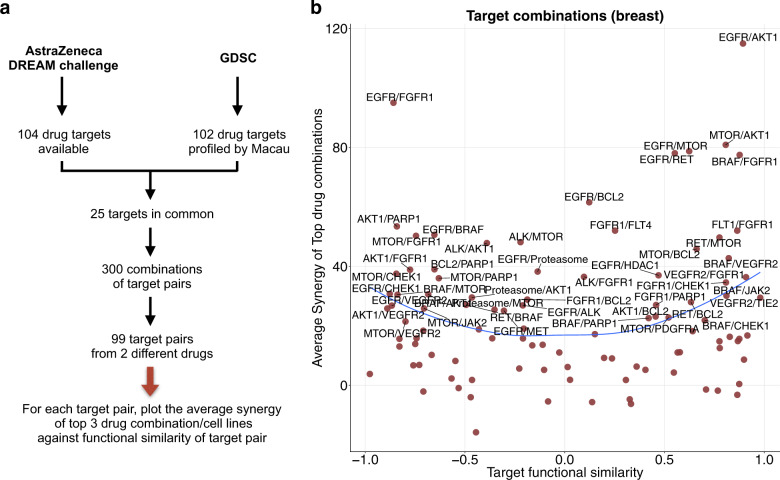
Influence of the similarity between target proteins on drug synergy. **a** We selected common targets from AstraZeneca DREAM and GDSC data sets. **b** The target functional similarity is the correlation between 2 targets by their interactions with the PROGENy pathways. A correlation of 1 implies that the activities of pathways correlates in the same way with drug efficacy on those proteins. A correlation of −1 implies opposite effects. The average synergy is computed for each target pair, as the mean of the top three synergistic drug-cell line pairs. We chose a threshold of 20 as a synergistic effect, and a score lower than −20 as an antagonistic effect, as in Menden et al.^16^. The blue line represents the loess smoothing.

To answer the question whether the functional similarity of drugs’ targets affect synergy, we used the AstraZeneca drug combination DREAM challenge data^16^, composed of 910 combinations and 85 cancer cell lines, which are also part of the GDSC panel. We selected the 25 target proteins from GDSC that are also part of the DREAM challenge data (Fig. 2a). There are 300 pairwise combinations from the *n* = 25 proteins, from which we selected 99 pairs where the two proteins are targeted by two different drugs in the GDSC panel, since we are interested in drug combinations. We considered the target pathway interaction matrix and for each combination of targets, we computed the Pearson’s correlation of the interaction score with the PROGENy pathways. The target combinations were then ranked from the most correlated pair to the most anticorrelated pair. For instance, the proteins BRAF and MEK are in the same pathway (ERK signaling), have a functional similarity of 0.74 (*P* = 0.0088) in skin cell lines, and are synergistic within this cancer type^17^. We consider that if the similarity between two target proteins is greater than 0.7 (Supplementary Fig. 7), then inhibition of the proteins triggers similar effects.

Synergy scores in AstraZeneca dataset are derived from the volume difference of an experimental five-by-five matrix (combinations tested at different drug concentrations) and the theoretical Loewe additivity surface inferred from both monotherapies (see “Methods”). To ascertain if target functional similarity can influence drug synergy, we chose the breast tissue as it is the most represented in the dataset (33 cell lines), and for each target pair, we plotted the observed average synergy scores of the top three synergistic drug1–drug2–cell triplets, against the target functional similarity of this target pair (Fig. 2b). We observed that synergy arises in both highly correlated and highly anti-correlated target groups (Fig. 2b). Remarkably, very few synergistic target pairs were found with a functional similarity close to zero (lowly correlated target group).

We tested the significance of our observation on breast (33 cell lines), colon (12 cell lines), and NSCLC (22 cell lines), by predicting the average observed synergy score of the top synergistic combinations using the absolute value of the target functional similarity (Supplementary Fig. 1). For breast, colon and lung tissues, the prediction performances (Pearson’s correlation) are *r* = 0.23, *r* = 0.36, and *r* = 0.17, respectively. The trend is stronger for colon compared to lung, which is why we chose to focus on colorectal cancer cell lines.

### External validation of the functional similarity metric

We further validated those trends on the NCI-ALMANAC dataset^18^. We used functional similarity from Sanger data to predict the synergy in NCI-ALMANAC. For 18 target pairs and 8 tissues in common, the prediction performance is *r* = 0.35 (Supplementary Fig. 2a), using the average functional similarity across all tissues to predict the average synergy. The small number of targets (18 versus 99 in AstraZeneca data) makes the average across tissues potentially more reliable than taking individual tissues. Although the performance is exceptional for brain and colon tissues, *r* = 0.75 and *r* = 0.32, respectively (Supplementary Fig. 2c, d), and failed for breast, *r* = −0.33.

These results made us hypothesize that target functional similarity based on pathway activations is a metric that can be used for compound prioritization: for any given target pair, the more functional similar or opposite two proteins are, the most likely synergy will arise. We reason that this could be due to complementary mechanisms of synergy that take place: *Mechanism 1 (Synergy by similarity)*: When two drugs have similar interaction profiles, they are most likely targeting some common mechanism. In this case, synergy may be achieved by double hit of the same pathway, or putatively inhibiting feedback loops. *Mechanism 2 (Synergy by compensation)*: In contrast, for functionally opposite proteins, when one pathway’s activation is correlated with drug sensitivity for targeting one protein, it is also correlated with resistance for targeting the other protein. This functional landscape may prevent the activation of compensatory escape pathways, and thereby increases the likelihood to observe synergy.

We developed a workflow for ranking synergy enrichment (Fig. 1a), based on multitask learning through the following steps: (i) Compute tissue specific interaction matrix between target proteins and pathway activities using single drug screening data. (ii) Find common target proteins between single drug screening data and drug synergy data. (iii) Compute pairwise Target functional similarity in single drug screening data for the selected common targets. (iv) Keep target pairs with absolute Target functional similarity greater than a certain threshold (e.g., 0.7). Our method returns a ranking of experimentally untested drug combinations from being likely to unlikely synergistic, which ultimately enables a prioritization for future experiments (Supplementary Data 1).

### Stratifying cancer cell lines for synergistic combinations

As an addition to the synergy prediction workflow, we propose a consecutive step to stratify cell lines from responders to nonresponders. For this, we use the inferred synergy mechanism and pathway activities of new samples to build specific models to predict synergy for new drug combinations. The synergy stratification workflow predicts the actual synergy scores on samples for a given target pair for which synergy has been described (either through experiments or from literature). For each of the previously described synergy mechanisms, we built specific models to predict synergy scores on new cancer cell lines (“Methods”, Fig. 1b). We used delta pathway activity (delta PA), a linear combination of pathway activity, single-nucleotide polymorphisms (SNP), and copy number variation (CNV) to predict synergy. Therefore, our models stratify the cell lines based on their “genomic context” (Methods). We only considered drug combination known to induce synergy, for the following reasons: (i) In practice, we decide about stratification only after knowledge of synergy potential. (ii) We predict synergy score with a linear combination of pathway activities (Methods), a relative concept that is not based on actual synergy scores, and therefore not designed to predict the existence of synergy.

We propose a general framework to predict synergy scores follows several key steps and we emphasize on the notion of “target combination” which represents the dual inhibition of two target proteins, regardless of the drugs that are used (Methods). We applied our methodology to AstraZeneca drug combination data for breast tissue and experimentally validated a predicted synergistic drug combination for colorectal cancer cell lines.

### Application to the AstraZeneca breast data set

We tested our synergy models on different target pairs by computing the Pearson’s correlation of observed versus predicted synergy scores on all available cell lines. The observed synergy is computed as the average of all drug combinations targeting a given target pair, across all available cell lines. Therefore, for each cell line, the observed average synergy may be computed for different drug combinations since the matrix of drug-cell line synergy is sparse.

We selected target pairs that fulfilled the following conditions: (1) Observed synergy score^19^ of top hits must be greater than 20, considered as a clear threshold for synergy^16^ (Fig. 2b). (2) Drug combinations have had to be tested in at least 10 cell lines, owing to the limitations of measuring performance by Pearson’s correlation. (3) At least two different drug combinations for the target pair were tested in each cell line, otherwise we excluded the cell line. We focused on the target pairs rather than specific drug pairs, in order to derive more robust insights.

**Fig. 3:**
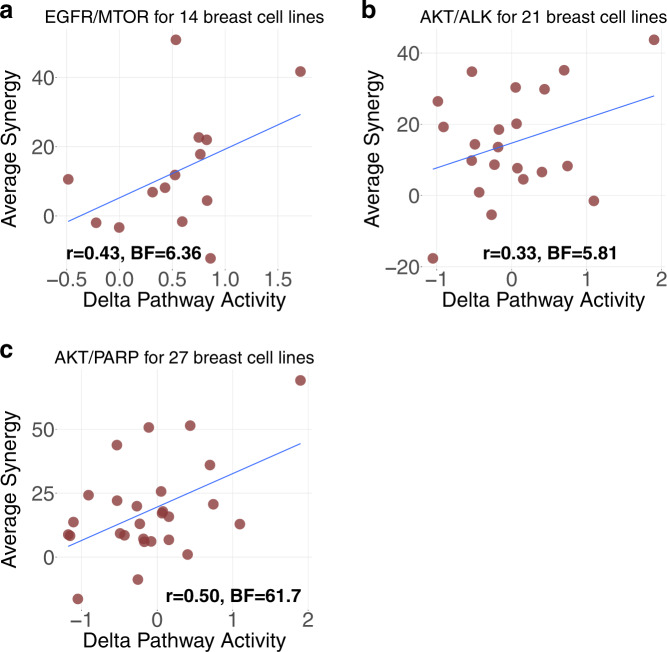
Prediction performance of drug synergy on breast tissue. **a**–**c** The prediction result for EGFR/MTOR, AKT/ALK, and AKT/PARP1 targets pairs on breast tissue, respectively (from AstraZeneca DREAM challenge data). Each dot represents a cell line; *y*-axis is the average observed synergy score of the top three drug1–drug2–cell line triplets; *x*-axis is the predicted synergy score (Delta Pathway Activity).

This leaves us with the following seven target pairs: AKT/EGFR, AKT/MTOR, BCL2/MTOR, EGFR/MTOR, AKT/BCL2, AKT/ALK, and AKT/PARP1, each representing several distinct drug combinations (3, 5, 3, 4, 4, 6, and 4, respectively). We applied our methodology on those target pairs (“Methods”, Supplementary Text 1), and obtained statistically significant prediction performances (Bayes Factor and *p* value of the Pearson’s correlation of observed versus predicted synergy scores, “Methods”) for the following pairs: EGFR/MTOR (*r* = 0.43, BF = 6.36, *p* = 0.12), AKT/ALK (*r* = 0.33, BF = 5.81, *p* = 0.21), and AKT/PARP1 (*r* = 0.50, BF = 61.7, *p* = 0.01) (Supplementary Table 1, Fig. 3). The average performance of all seven pairs is *r* = 0.27 using Leave One Out Cross Validation (“Methods”, Supplementary Table 1).

Overall, among all target pairs, EGFR/MTOR was predicted with Model 1 (synergy by similarity). AKT/ALK and AKT/PARP1 were predicted using Model 2 (synergy by dissimilarity).

### Independent experimental validation on colorectal cancer cell lines

We then attempted to experimentally validate our synergy stratification workflow in colorectal cancer. In order to ascertain our method’s capability to detect synergy, we chose a drug combination in the following way:

(i) We focused on drug combinations involving the protein BRAF in colorectal cancer, which is frequently mutated in this cancer type (~10% of The Cancer Genome Atlas (TCGA) patients^20^) and can result in uncontrolled, non-EGFR-dependent cellular proliferation^21^. Despite the success of BRAF inhibitors in melanoma, in colorectal cancer BRAF monotherapies largely fail to demonstrate clinical efficacy in BRAF^V600^-mutants due to feedback activation of EGFR^22,23^. BRAF^V600^ is the most common mutation for BRAF (90% cases) where valine is substituted by glutamate in the codon 600. Such mutation can lead to a 500-fold increased activation, stimulating the constitutive activation of MEK/ERK pathway in tumor cells^24^. Thus, there is a need for novel combination therapies^25–27^ with BRAF inhibitors in colorectal cancer.

(ii) We exclude combinations that target, on average, more than three proteins, since too many targets can render the model less precise. There are 101 targets in GDSC besides BRAF, and we computed their functional similarities with BRAF. Insulin receptor (IR) ranked first with a target functional similarity of 0.73 for the BRAF/IR pair. Insulin has been described as promoting cell proliferation in colorectal cancer by activating MAPK signaling^28^, which could explain a similarity with BRAF. Therefore, we chose BRAF/IR as a candidate for validation. We used Dabrafenib as a BRAF inhibitor and BMS-754807 as a selective inhibitor of IR^29^.

(iii) Since BMS-754807 also targets insulin growth factor 1 receptor (IGF1R), we chose the proteins BRAF, IR, and IGF1R as drug targets and derived the Delta PA to predict synergy (“Methods”, Supplementary Fig. 4H, Supplementary Data 1).

We experimentally validated our methodology on newly generated combination screenings with Dabrafenib and BMS-754807 on 48 colorectal cancer cell lines from the GDSC panel. The synergy score is computed with DeltaXMID (Methods). The Pearson’s correlation of observed versus predicted synergy score is 0.31 for all 48 cell lines (“Methods”, Supplementary Data 1, Fig. 4a).

**Fig. 4:**
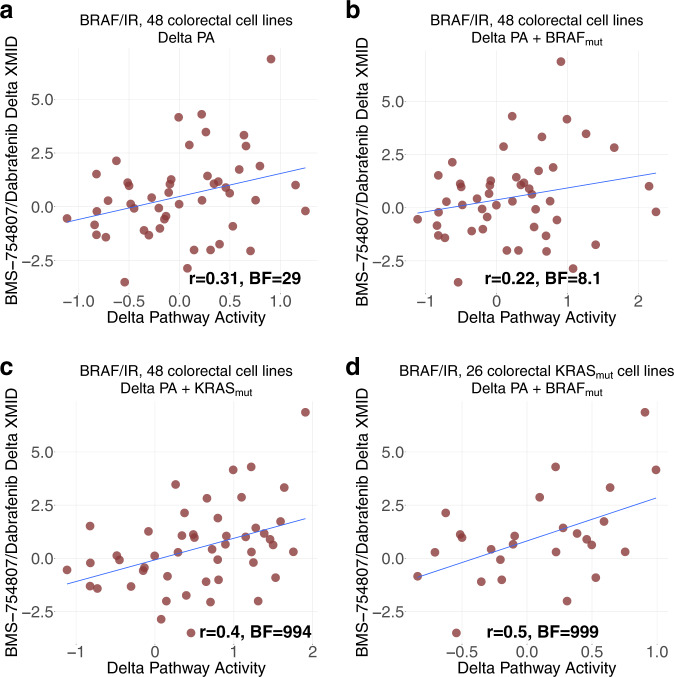
Prediction of BRAF/IR synergy on colorectal tissue. **a** Prediction result of BRAF/IR (BMS-754807/Dabrafenib) on all 48 colorectal cancer cell lines. **b** Result with BRAF status included in Delta PA formula. **c** Result with KRAS status included in Delta PA formula. **d** Result on the subset of 26 KRAS_mut_ colorectal cancer cell lines with BRAF status included in Delta PA formula.

We further reasoned that inclusion of information about top predictive pathways should increase the predictive power. In the case of the Dabrafenib and BMS-754807, the most predictive pathway is Hypoxia (Methods). KRAS mutation has been shown to differentially regulate the hypoxic induction of HIF-1α and HIF-2α in colon cancer^30^. Hence, for each drug combination, we computed the Delta Pathway activities as a predicted synergy. This is a vector of values with dimension the number of cell lines that are concerned. To include the KRAS mutational status, we simply add a binarised variable for wild-type and mutant KRAS cell lines in addition to the previously computed scores. The prediction performance rose to 0.4 (Fig. 4c). Accordingly, adding BRAF mutation did not improve the performance (Fig. 4b), as MAPK was not as highly ranked as Hypoxia pathway for this combination. However, the performance rose to *r* = 0.5 (BF = 999, *p* < 2.2e−16) by including BRAF status in the delta PA formula for the subset of 26 KRAS mutant cell lines (Fig. 4d), suggesting an effect of BRAF mutations only when KRAS is mutated. This can have clinical relevance for around 1% of TCGA colorectal cancer patients have both KRAS and BRAF mutated^20^.

### Comparison with supervised learning approaches

Supervised learning has long dominated the prediction of drug pharmacological effect. However, its intrinsic reliance on training data makes it difficult to predict in a data-sparse situation. Furthermore, the prediction itself generally does not bring any biological insights (Supplementary Table 1). Having these differences in mind, we performed a comparison with our hypothesis based method for synergy stratification (Supplementary Table 2).

In the AstraZeneca DREAM challenge, an ensemble of best performing models was trained on the AstraZeneca DREAM combinatorial data, and consecutively tested on an independent combinatorial screen from Merck^31^, which achieved a weighted mean correlation of 0.15–0.17. In comparison, we used our synergy stratification workflow on the GDSC panel for hypothesis generation and the AstraZeneca dataset for testing, considered the setting of predicting synergy of new drugs on new cell lines (Supplementary Fig. 5). For the seven target pairs (29 drug combinations) from breast tissue, we were able to reach an average drug-wise correlation of 0.27. Of note, the two methods are of very different nature and have very different applications. Therefore, prediction performances should not be compared directly. In the DREAM challenge, synergy scores of drugs/samples are predicted without any prior knowledge of whether a drug combination leads to synergy or not. In contrast, in our synergy stratification workflow, we assumed a synergy potential and consecutively predict the stratification based on pathway activity and mutational profiles.

## Discussion

In this paper, we presented an approach with two workflows, one for drug synergy prediction and one for synergy stratification. Our approach only requires basal gene expression, drug-response (IC50) of monotherapy, and information on drug targets, to prioritize drug combinations. In contrast to most prediction methods, neither information on synergy of drug combinations, nor on the effects of single agents on gene expression, is needed. The synergy prediction workflow can be a powerful framework for compound prioritization in large scale drug screenings. For instance, only testing drugs targeting two functionally very similar or very opposite proteins (|correlation| > 0.7) could significantly reduce the search space, therefore decreasing the cost of drug combination screens. We validated our result on the NCI-ALMANAC dataset^18^. Our other workflow for synergy could potentially be used to maximize the drug efficacy of drugs already known to induce synergy, by choosing on which cells (and eventually patients) to apply them. Indeed, knowing that a pair of compounds can be synergistic does not tell us on which patients it will occur. As real world use case, we envision that for any drug combination described as synergistic, this method could potentially inform about the subset of patients most likely to benefit, based on their transcriptomics profiles, provided enough good quality data to apply the methods.

We introduced the notion of functional similarity between two target proteins. This metric sheds lights on two scenarios where drug synergy occurs: when drugs are targeting functionally similar proteins (EGFR/MTOR) and when they are targeting functionally opposite proteins (AKT/ALK and AKT/PARP1). We hypothesize that combinations of functionally similar targets may lead to synergy due to inhibition of compensatory feedback loops and/or increases target inhibition, whilst functionally dissimilar targets are more likely to be synergistic by targeting potential escape mechanisms. Our results support that synergy occurs and is much easier predicted when the targets are functionally very similar or very opposite. Portraying the interaction between target proteins and pathway activities allowed us to recognize the different synergy cases. We then built models by leveraging interpretable gene expression signature (i.e., pathway activity scores) as biomarkers of synergy, and thereby increase potency and reduce the risk of toxicities in humans. Our method enables to stratify responders within a tissue type based on the genomic and transcriptomic profile. Based on that, we applied our method to seven target pairs (representing 29 drug combinations) in breast cancer cell lines. Finally, we predicted and validated a drug combination synergy (Dabrafenib/BMS-754807) on 48 colorectal cancer cell lines.

There are several limitations to this study that can be the focus of future work: (i) The synergy models are simple and could be extended to take into account non-linear effects of pathways adding coefficients to each pathway and including logic (AND/OR) gates. But this would require an extensive training set. (ii) In this present work, target functional similarity is defined with respect to 11 PROGENy pathways, which do not necessarily capture all cancer mechanisms. Besides, only a few cell lines have been used for perturbation experiments to represent each tissue, which does not necessarily capture the whole complexity of the cancer specific signaling mechanism. Expanding this geneset to include more pathways, as well as using more cell lines for each cancer type is likely to improve our models. (iii) In order to predict synergy of new compounds, drug targets have to be profiled by large scale monotherapy drug screening experiments across hundreds of cell lines. Thus, to increase the space of combinations, we require the corresponding monotherapy data. This cost provides important gains, as cost for monotherapy grows linearly, but exponentially for combinations. (iv) There is currently limited tissue specific publicly available drug synergy data. Such datasets could be highly valuable to further refine our and other approaches.

Our study findings are aligned to those of the DREAM drug combination challenge^7^, where synergy was found to be highly context dependent. In our case, we predicted synergy with a linear combination of pathway activities. Bansal et al.^7^ predicted synergy from single-compound perturbation data. They found that synergy occurs for drug pairs which induce very similar or very opposite gene perturbation statuses. We used single-compound drug response data and the Macau algorithm to compute the target functional similarity, which reflects the similarity of drug response changes for different pathways after targeting a specific protein. We found that compounds that have very similar or very opposite functional profiles tend to be more synergistic. We used the inferred synergy mechanism and pathway activities to predict synergy of new compounds.

We compared our synergy stratification workflow with state-of-the-art supervised learning (Supplementary Text 2, Supplementary Table 1) and highlighted the pros and cons for each methodology: (i) Naive supervised learning approaches are easy to implement, do not require extensive domain expertize (although still highly valuable), and can be used for all possible prediction settings (Supplementary Fig. 5). However, they require an extensive set of drug combination drug response data as training set. (ii) For our synergy stratification methodology, linear combination of pathway activities is well suited for biological interpretation. However, it can only be used in drug-wise setting and requires significant domain knowledge and literature evidence.

In terms of translatability to the in vivo setting, supervised learning needs combination data of the organism of interest for training, whereas our method requires the target functional similarity to be built based on monotherapy data on the organism of interest. We attempted to test our methodology to Novartis PDX data^32^, but it was not possible due to the fact that the drug targets and gene expression were not predictive of the single drug response, with prediction performance at random level. Therefore, we could not derive a robust and reliable interaction matrix/Target functional similarity from this data^15^. The random performance of single drug response prediction on Novartis PDX data^32^ might be explained by (i) the high level of missing values of the drug response matrix (64%), (ii) the model system could be so complex that transcriptomics alone does not reflect the underlying biology enough to be predictive of drug response, and (iii) that measurements could have been inaccurate, owing to a lack of standardization for this new technology. When larger and more mature datasets of this kind become available, it will be possible to test our approach on such an in vivo context.

Palmer and Sorger^33^ stated that successful drug combination in tumor shrinkage are mostly due to targeting unrelated pathways, without any real synergy. They define drug action similarity by the correlation of single drug response data, which resembles our use of target—pathway based similarity score. They concluded that highly correlated independent drug responses can explain the majority of combination clinical trial (synergy), whereas lowly correlated independent drug responses makes independent action of drugs the dominant mechanism in clinical populations (additivity). While the analysis is different as we used cell line data, there are commonalities in our findings.

Our method focuses on the putative drug target, whilst many compounds may target multiple proteins and in particular at high drug concentrations off-target effects are pervasive. Notably, our method is generalizable to multiple targets. We can consider all targets’ interaction values with the pathways and correct for different affinities of the drugs for each putative target, as well as the significance of the interaction values. More complex modeling will be necessary as synergy can arise from different target pairs, as well as multiple hypothesis testing will pose another challenge.

In summary, exploring the interactions between drug targets and signaling pathways in a tissue specific manner can provide a novel in-depth view of cellular mechanisms and drug modes of action, which can ultimately rationalize drug combination strategies in cancer. Target functional similarity could be used as a metric for compound prioritization. Synergy by similarity hypothesis could be a rational for first-line treatment, while synergy by opposite effect could potentially fit patients having acquired resistance.

## Methods

### Matrix factorization with Macau

Macau trains a Bayesian model for collaborative filtering by also incorporating side information on rows and/or columns to improve the accuracy of the predictions^14^ (Fig. 1a). Drug response matrix (IC50) can be predicted using side information from both drugs and cell lines. We use target protein as drug side information and transcriptomics/pathway as cell line side information. Each side information matrix is then transformed into a matrix of L latent dimension by a link matrix. Drug response is then computed by a matrix multiplication of the two latent matrices. Macau employs Gibbs sampling to sample both the latent vectors and the link matrix, which connects the side information to the latent vectors. It supports high dimensional side information (e.g., millions of features) by using conjugate gradient based noise injection sampler.

### Pathway activities

We transformed the transcriptomics data into pathway activity scores using PROGENy (Fig. 1a, Supplementary Fig. 8). PROGENy is a data driven pathway method aiming at summarizing high dimensional transcriptomics data into a small set of pathway activities. PROGENy leverages hundreds of publicly available gene expression perturbation experiments. In the chosen experiments, cancer cell lines have been treated by a perturbation agent which activates or inhibits one of the PROGENy pathways. After computing the gene expression *z*-scores of the Perturbed—Control, we fit a multiple linear model of the *z*-scores as a function of the pathway statuses. The *z*-scores represents the change in gene expression, and we aim at determining the role of the pathway activation statuses in this change. We derive a weight matrix, which is the contribution of each pathway for the gene’s expression changes. For new gene expression samples where we want to estimate the pathway activities, we multiply the gene expression matrix with the weight matrix. Therefore, for a given sample, the activity score of a certain pathway is the sum for all genes involved in this pathway’s signature, of the product of the gene’s expression by its weight. PROGENy derives pathway signatures from the genes that are altered when perturbing a pathway instead of solely from the genes within the pathway as described in literatures. Schubert et al.^12^ found that the most representative genes of a pathway in the PROGENy weight matrix are often not the pathway components themselves.

This improves the estimation of pathway activities compared to knowledge based gene sets^12^. We used the following 11 PROGENy pathways: EGFR, NFkB, TGFb, MAPK, p53, TNFa, PI3K, VEGF, Hypoxia, Trail, and JAK-STAT.

### Interactions between drug target and pathway activities

The core result from which we derive the target functional similarity is the interaction matrix between drug targets and pathway activities. We computed those interactions between target proteins and signaling pathway activation status with respect to drug response (IC50) by multiplying the 2 link matrices β_D_ and β_C_ and averaging across 600 Gibbs samplings (Fig. 1a). We then repeated this process 40 times and took the average value. This interaction can be defined as the importance for those two entities to be simultaneously involved in order to have an impact on drug response^15^, e.g., how the simultaneous activation of a certain pathway and targeting a certain protein can be associated with drug response. For instance, a strong interaction between protein MEK1/MEK2 and pathway EGFR in pancreatic cancer is interpreted as follows: activation of the EGFR pathway correlates with sensitivity when targeting MEK1/MEK2. If this were a causal relationship, it could mean that EGFR pathway activation confers sensitivity to any drug targeting protein MEK1/MEK2.

We used the GDSC cell line panel that contains drug response (IC50) data of 265 drugs on 990 cell lines. For each of the 16 tissues (with more than 20 cell lines), we computed the interaction matrix between drug targets and pathway activities using the multitask learning algorithm Macau^14,15^. Our algorithm tries to learn multiple tasks (predicting multiple drugs) simultaneously and uncovers the common (latent) features that can benefit each individual learning task^34^. We used manually curated target proteins for the drug (Supplementary Data 3), and gene expression derived pathway scores for the cell lines. The interaction matrix gives hints about the drug’s mode of action, by uncovering in which condition (pathway status) targeting a certain protein correlates with higher drug sensitivity.

To assess the robustness of the prediction, we produced 40 interaction matrices and randomly selected 36 to take the average and repeated this process 50 times. The average correlation between any pair of matrices from a random draw among those 50 matrices is *r* = 0.998 (sd = 0.0005). We next sought to determine how much the functional similarity varies based on the interaction matrices. We randomly select two protein targets and computed their functional similarity across the 50 interactions matrices, and repeated this experiment 1000 times. For target pairs with a similarity greater than 0.4 (0.7), the average coefficient of variation of the functional similarity is 7% (3%). We deem that a coefficient of variation around 5% for a single pair is reasonable since we use the functional similarity to generate hypotheses and not for actual predictions. Finally, we tested the correlation between the functional similarities of the selected target pairs with the drug synergy score as shown in Supplementary Fig. 1. The mean correlation across 50 interaction matrices is 0.22 (sd = 0.014) for breast and 0.45 (sd = 0.06) for colon tissue.

### Significance of the synergy prediction result

In order to assess the significance of the predicted synergy on breast and colorectal tissues (Supplementary Fig. 1), we estimate *p* values by simulating random data. Therefore, we randomly shuffled the drug combination labels for each tissue 1000 times and generated a null distribution of no synergy. We defined the *p* value as the number of times our model’s correlation is greater or smaller (two-tailed test) than the random models expected distribution of correlations divided by 1000. For reference values of *r* = 0.23 (breast) and *r* = 0.36 (colon), the corresponding *p* values are *p* = 0.065 and *p* = 0.045, respectively.

We then estimated a Bayes Factor (BF) to compare the performance of the model on each tissue based on 1000 random samples. Specifically, the BF is calculated as the ratio of number of samples on which the reference performance is better than the null model and the number of samples on which the null model is worse than the reference performance. The choice of this metric is that it is independent of the sample size. For reference values of *r* = 0.23 (breast) and *r* = 0.36 (colon), the BF are 21.7, and 11.1, considered strong evidence for both.

### Significance of the synergy stratification result

To assess the synergy stratification result from AstraZeneca breast dataset (Fig. 3), we randomly shuffled the cell lines labels independently for each drug combination following the same procedure. The two-tailed *p* values are: *p* = 0.12 (EGFR/MTOR), *p* = 0.21 (AKT/ALK), and *p* = 0.01 (AKT/PARP1). All *p* values are significant at false-discovery rate (FDR) < 25%. The BF are 6.36 (EGFR/MTOR), 5.81 (AKT/ALK), and 61.7 (AKT/PARP). We used the same procedure to assess the significance of synergy stratification on 48 colorectal cancer cell lines (Fig. 4), the *p* values are: *p* = 0.15 (Fig. 4a), *p* = 0.13 (Fig. 4b), *p* = 0.01 (Fig. 4c), and *p* < 2.2e−16 (Fig. 4d). All *p* values are significant at FDR < 25%. The BF are: 29 (Fig. 4a), 8.1 (Fig. 4b), 994 (Fig. 4c), and 999 (Fig. 4d).

### Building specific models for each synergy hypothesis

The core idea of the synergy stratification workflow is to predict the synergy score using the pathway context in addition to drug target information. We believe that synergy is not only due to the drugs’ properties, but also dependant on the signaling context.

#### Synergy Model 1 (maximizing drug sensitivity)

For functionally similar target pairs (Mechanism 1), we rank the pathways based on their sensitive or resistant interaction profile with respect to the drug targets. We postulate that synergy is maximized under a pathway condition where both drugs’ effects are maximized. The optimal condition for synergy is therefore when pathways associated with drug sensitivity are upregulated, and pathways associated with drug resistance are downregulated (Supplementary Fig. 3). As a consequence, if two target proteins have strong functional similarity, e.g., high correlation between their interaction profile with pathway activities, synergy is maximized when the sensitizing pathways are activated and pathways conferring resistance not activated. We predict synergy by taking the average of the top N sensitive pathway scores, subtracted by the average of the top M resistant pathway scores. Therefore, for each cell line, we introduce the concept of delta PA to predict synergy:

1 $${\mathrm{Delta}}\,{\mathrm{PA}} = \frac{{\mathop {\sum}\nolimits_1^{\mathrm{N}} {({\mathrm{Top}}\,{\mathrm{sensitive}}\,{\mathrm{pathways}})} }}{N} - \frac{{\mathop {\sum}\nolimits_1^{\mathrm{M}} {\left( {{\mathrm{Top}}\,{\mathrm{resistant}}\,{\mathrm{pathways}}} \right)} }}{M} \pm {\mathrm{genomics}}.$$

We compute the average pathway score for both sensitive and resistant groups. Each group should include a minimum of one to a maximum of three pathways. We select the top pathways with group membership thresholds determined by cross validation. If applicable, we include in the formula the genomic information which can be mutation (SNP) or CNV. For instance if protein EGFR is targeted, we include CNV_EGFR_. Group membership parameters are defined using cross-validation.

#### Synergy Model 2 (maximizing drug resistance)

For functionally opposite target pairs (Mechanism 2), when a pathway’s activation is associated with resistance for one target protein, it is also associated with sensitivity for the other target protein, to compensate. Two drugs can be individually ineffective, but more effective when combined. Therefore, synergy may arise in a situation of drug resistance. This could be explained by the fact that if a cell line is resistant for one (or both) of the drugs, there is “more opportunities” to be synergistic. When both drugs kill a given cell very efficiently, there is no synergy, as both drug A alone, drug B alone and combination A + B can kill all the cells. Unsurprisingly, resistance biomarkers were found to be predictive of synergy in the recent AstraZeneca DREAM challenge^16^. Therefore Delta PA should maximize the pathways conferring resistance and minimize the sensitizing pathways. The formula becomes

2 $${\mathrm{Delta}}\,{\mathrm{PA}} = \frac{{\mathop {\sum}\nolimits_1^{\mathrm{M}} {({\mathrm{Top}}\,{\mathrm{resistant}}\,{\mathrm{pathways}})} }}{M} - \frac{{\mathop {\sum}\nolimits_1^{\mathrm{N}} {({\mathrm{Top}}\,{\mathrm{sensitive}}\,{\mathrm{pathways}})} }}{N} \pm {\mathrm{genomics}}.$$

Model 2 is less likely to suit functionally similar pairs (Mechanism 1). If the two drugs have similar functional profile, maximizing the resistance scenario equals increasing the dose of the same inefficient drug, thus, unlikely to improve the outcome. Likewise, Model 1 is less suitable for Mechanism 2, as maximizing the sensitizing pathways is the same as prioritizing a situation where drug 1’s sensitive effect outweighs drug 2’s resistant effect. Thus, Mechanism 2’s core idea would become obsolete, as by definition, the resistance scenario must prevail in case of escape mechanism. Of note, having an opposite functional profile does not imply Mechanism 2. An opposite pathway-response profile for two targets, offers the “functional scenario” for the cell to escape the damage induced by one drug. Yet, there could still be a scenario which maximizes the sensitizing pathways. This corresponds to two drugs targeting completely independent pathways, which is more due to independent actions rather than additivity or synergy^33^.

### General framework for predicting synergy score

*Step 1*: For two given target proteins T1 and T2, find their interactions with the PROGENy pathways using Macau (Supplementary Fig. 4).

*Step 2A*: If available, use literature to guide the choice of Model, e.g., if we know that a drug combination is synergistic when a pathway X is activated, the model would be the one which gives a positive sign for pathway X. Otherwise go to *Step 2B*.

*Step 2B*: Compute the functional similarity between T1 and T2 (Pearson’s correlation between T1 and T2’s interactions with the pathways).

If the correlation is close to 1, use *Model 1* to define the Delta PA formula.

If the correlation is close to −1, use *Model 2* to define the Delta PA formula.

If the correlation is between −0.4 and 0.4, it is an undetermined case.

*Step 3*: Find top sensitive and top resistant pathways (as previously described in synergy models). Take into account literature evidence in choice of pathways (for known drugs or targets). If a pathway is described as important in literature but does not appear in top three of a group, we include it, as well as any pathway separating the first from the one of interest, while respecting the limit of three pathways per group.

*Step 4*: In case of multiple drugs representing the same target pair, as in the AstraZeneca DREAM data set, keep the drugs that are specifically targeting the two proteins of interests, while removing those having off target effects (at least 3 drug pairs left).

*Step 5*: Use the Delta PA formula to predict synergy of a drug combination targeting T1 and T2. The pathway activities of the formula are computed by PROGENy on the cell lines of interest.

More details about the cross validation procedure and synergy score computation are in Supplementary Methods and Supplementary Fig. 6.

## Supplementary information

Supplementary information

nr-reporting-summary

Supplementary Data 1

Supplementary Data 2

Supplementary Data 3

## Data Availability

GDSC data were downloaded from: http://www.cancerrxgene.org/. Drug IC50 version 17a, basal gene expression 12/06/2013 version 2, drug target version March 2017. DREAM drug combination challenge data were acquired through an AstraZeneca Open Innovation Proposal. NCI-ALMANAC data are downloaded from the publication Holbeck et al.^18^. Novartis PDX data are downloaded from the publication Gao et al.^32^.

## Code availability

The source code to generate the interaction matrices is available at: https://github.com/saezlab/Macau_project_1. The source code of the result analysis is available at: https://github.com/saezlab/Macau_Synergy_Prediction.
