# Supplementary material for: Stratification and prediction of drug synergy based on target functional similarity
Source: NPJ Syst Biol Appl. 2020 Jun 2;6:16. doi: 10.1038/s41540-020-0136-x (PMC7265486; doi:10.1038/s41540-020-0136-x)
Supplement: Supplementary file 1 — Supplementary information [file 41540_2020_136_MOESM1_ESM.pdf]

Supplementary files to

Stratification and prediction of drug synergy based on target functional similarity

Mi Yang[1,5,9], Patricia Jaaks[3], Jonathan Dry[2], Mathew Garnett[3], Michael P. Menden[6,7,8,#], Julio Saez-Rodriguez[4,5,*,#]

[1]Heidelberg University, Faculty of Biosciences, Germany.
[2]Oncology, IMED Biotech Unit, AstraZeneca R&D Boston, Waltham MA, USA.
[3]Wellcome Trust Sanger Institute, Wellcome Trust Genome Campus, Cambridge CB10 1SA, UK.
[4]Institute for Computational Biomedicine, Heidelberg University Hospital and Heidelberg University, Faculty of Medicine, Bioquant Heidelberg, Germany.
[5]RWTH Aachen University, Faculty of Medicine, Joint Research Centre for Computational Biomedicine (JRC-COMBINE), Aachen, Germany.
[6]Institute of Computational Biology, Helmholtz Zentrum München - German Research Centre for Environmental Health, 85764 Munich, Germany.
[7]Department of Biology, Ludwig-Maximilians University Munich, 82152, Martinsried, Germany
[8]German Centre for Diabetes Research (DZD e.V.), 85764, Neuherberg, Germany
[9]Current affiliation: Division of Oncology, Department of Medicine, Stanford Cancer Institute, Stanford University, Stanford, CA, USA

[#] co-senior
[*]Corresponding author: Julio Saez Rodriguez, Institute for Computational Biomedicine, Im Neuenheimer Feld 267 (BioQuant, BQ 0053) 69120 Heidelberg, Germany, julio.saez@bioquant.uni-heidelberg.de

SUPPLEMENTARY DATA

Supplementary Methods

Cross validation of group membership thresholds

Prediction using a fixed threshold for both groups

For computing the Delta Pathway Activity formula, we chose the group membership to be the same in the top sensitive and top resistant groups. We only included pathways producing more than 70% of the effect of the first selected pathway. This choice of parameter seems to be a reasonable choice between including no additional pathway (threshold close to 100%) and including too many potentially irrelevant pathways (50%). We did a sensitivity analysis for this parameter and used different thresholds for the two different groups (sensitive and resistant). We fixed one group’s threshold while varying the other group’s threshold. We observe that for many target pairs, the sensitive group’s threshold is rather stable (**Supplementary Fig. S6**). For the sensitive group, the model is quite robust to variation of this parameter, whereas for the resistant group, a lower threshold (including more pathways) seems to yield better result. This could be explained by the fact that GDSC drug screening adjusts the drug concentration so that only a few cell lines respond while most are resistants. Therefore, more information are reflected from the resistant side than from the sensitive side.

Prediction using cross validated and different thresholds for each group

We next predicted each target pairs of AstraZeneca breast data with a Leave One Out Cross Validation (LOOCV) to optimize the group membership thresholds. For each target pair, we used as thresholds the best values all target pair of the training set. The average prediction of the 7 target pairs is 0.27. Finally, we used the average parameters for the breast data to predict the BRAF/IR in colon data (**Supplementary Table S1**).

Drug synergy metrics

For AstraZeneca dataset, drug effects on cancer cell lines are measured at several concentrations for each drug in a 5-by-5 matrix format. Therefore, the effect is described by a dose-response surface rather than a curve. The benefit of a drug combination can be partly assessed by the extra effect obtained when combining the drugs. Drug combinations are classified as synergistic, additive or antagonistic, based on the deviation of the observed drug combination response from the theoretical additive response. The theoretical additive response is quantified with the Loewe additivity model[1–4] based on the monotherapies of both drugs. Loewe additivity assumes the two drugs act on a protein through a similar mechanism. Synergy score is quantified with Combenefit[5], which calculates the volume between the experimentally observed on theoretical response surface. As in the AstraZeneca DREAM challenge, we consider a score greater than 20 as synergistic[6].

In colorectal cancer, we tested the drug combination of BMS-754807 and dabrafenib in 48 colorectal cancer cell lines. BMS-754807 (S2807, Selleckchem) was screened at 0.5 µM

against a 7 point dose response of dabrafenib (S1124, Selleckchem), ranging from 10 nM- 10 µM. The XMID, which is akin to an IC50, of dabrafenib alone and dabrafenib in combination with BMS-754807 were calculated and the ΔXMID=XMID(dabrafenib)-XMID(dabrafenib+BMS-754807) calculated. The fold difference in XMID can be calculated by y-fold=2^ΔXMID, hence a ΔXMID of 3.32 corresponds to a 10-fold lower XMID for dabrafenib + BMS-754807 compared to dabrafenib alone.

Supplementary text: Methodology applied to breast tissue

We explained through target-pathway interactions, two mechanisms of drug synergy. In order to validate our synergy models, we first looked at public data, using the DREAM AstraZeneca drug combination challenge[6], which experimentally tested >120 folds drug combinations compared to the previous Bansal et al. challenge. Furthermore, the AstraZeneca challenge expanded the number of tested cell lines including their deep molecular characterisation enabling for the first time identification of synergy biomarkers. We tested our model on 7 target pairs (29 drug combinations) from the AstraZeneca DREAM challenge[6], and chose breast as the most represented tissue with 33 cell lines.

We applied our general framework to predict synergy scores. The first step was to determine the top sensitive and top resistant pathways for a certain target - pathway pair (**Supplementary Figure 4**). We then derived the formula of Delta Pathway Activity and predicted the drug synergy (**Supplementary Table 1**). When choosing between Model 1 and 2 for the synergy model, the target functional similarity was the main criteria. If the similarity is close to 1, we use Model 1. If the similarity is close to -1, we use Model 2.

PI3K/AKT/MTOR pathway plays a significant role in treatment resistance in breast cancer[7]. Therefore, we hypothesized that the PI3K pathway will be informative of the synergy if AKT is targeted. Therefore, each time AKT is targeted, we included PI3K pathway as well as any pathway between the first one and PI3K, while respecting the limit of maximum three pathways per group.

When grouping pathways in the top sensitive and top resistant groups, we consider only those that have at least one significant interaction with the drug targets. If not significant, we discard the pathway. Exceptions are made when only one pathway is included (the top sensitive or top resistant one) and when the pathway has a stronger interaction than a pathway included by prior knowledge (literature).

For AKT/EGFR (**Supplementary Figure 4A, Fig. 3A**): the top sensitive pathway is EGFR and the top resistant are MAPK. The target functional similarity between AKT1/2 and EGFR is 0.9 (**Supplementary Table 1**). Therefore, we used synergy Model 1. Since protein EGFR is targeted, we also added CNV information:

$$Delta\ PA\ (AKT/EGFR)_{breast} = \frac{EGFR\ +\ NFkB\ +\ PI3K}{3} - MAPK + CNV_{EGFR}$$

For AKT/MTOR (**Supplementary Fig. S4B, Fig. 3B**): the top sensitive pathways are EGFR and VEGF. The top resistant pathways are MAPK and TNFa. The target functional similarity

between AKT1/2 and MTOR is 0.8 (**Supplementary Table S1**). Therefore, we used synergy Model 1:

$$Delta\,PA\,(AKT/MTOR)_{breast} = \frac{EGFR + VEGF + PI3K}{3} - \frac{MAPK + TNFa}{2}$$

For BCL2/MTOR (**Supplementary Fig. S4C, Fig. 3C**): the top sensitive pathways are VEGF, NFkB and Trail and the top resistant pathways are MAPK and TNFa. The target functional similarity between BCL2 and MTOR is 0.7 (**Supplementary Table S1**). Therefore, we used synergy Model 1:

$$Delta\,PA\,(BCL2/MTOR)_{breast} = \frac{VEGF + NFkB + Trail}{3} + \frac{MAPK + TNFa}{2}$$

For EGFR/MTOR (**Supplementary Fig. S4D, Fig. 3D**): the top sensitive pathways are EGFR and NFkB. The top resistant are MAPK and TNFa. The target functional similarity between EGFR and MTOR is 0.6 (**Supplementary Table S1**). Therefore, we used synergy Model 1. Since protein EGFR is targeted, we also added CNV information:

$$Delta\,PA\,(EGFR/MTOR)_{breast} = \frac{EGFR + NFkB}{2} - \frac{MAPK + TNFa}{2} + CNV_{EGFR}$$

For AKT/BCL2 (**Supplementary Fig. S4E, Fig. 3E**): the top sensitive pathway is EGFR and the top resistant pathway is MAPK. The correlation between AKT1/2 and BCL2 is 0.5 (**Supplementary Table S1**). In this case, we used Model 1:

$$Delta\,PA\,(AKT/BCL2)_{breast} = \frac{EGFR + VEGF + PI3K}{3} - MAPK$$

For AKT/ALK (**Supplementary Fig. S4F, Fig. 3F**): the top sensitive pathway is EGFR and the top resistant pathways are MAPK and TNFa. The target functional similarity between AKT1/2 and ALK is -0.4 (**Supplementary Table S1**). Therefore, we used synergy Model 2:

$$Delta\,PA\,(AKT/ALK)_{breast} = \frac{MAPK + TNFa}{2} - \frac{EGFR + VEGF + PI3K}{3}$$

For AKT/PARP1 (**Supplementary Fig. S4G, Fig. 3G**): the top sensitive pathway is EGFR and the top resistant pathways are MAPK and TNFa. The correlation between AKT1/2 and PARP1 is -0.8 (**Supplementary Table S1**). In this case, we used Model 2:

$$Delta\,PA\,(AKT/PARP1)_{breast} = \frac{MAPK + TNFa}{2} - \frac{EGFR + VEGF + PI3K}{3}$$

References

1. Loewe, S. The problem of synergism and antagonism of combined drugs. *Arzneimittelforschung* **3**, 285–290 (1953).
2. Berenbaum, M. C. What is synergy? *Pharmacol. Rev.* **41**, 93–141 (1989).
3. Loewe, S. Die quantitativen Probleme der Pharmakologie. *Ergeb. Physiol.* **27**, 47–187 (1928).
4. Fitzgerald, J. B., Schoeberl, B., Nielsen, U. B. & Sorger, P. K. Systems biology and combination therapy in the quest for clinical efficacy. *Nat. Chem. Biol.* **2**, 458–466 (2006).
5. Di Veroli, G. Y. *et al.* Combenefit: an interactive platform for the analysis and visualization of drug combinations. *Bioinformatics* **32**, 2866–2868 (2016).
6. Menden, M. P. *et al.* Community assessment of cancer drug combination screens identifies strategies for synergy prediction. (2017) doi:10.1101/200451.
7. Paplomata, E. & O'Regan, R. The PI3K/AKT/mTOR pathway in breast cancer: targets, trials and biomarkers. *Ther. Adv. Med. Oncol.* **6**, 154–166 (2014).

Supplementary tables

Supplementary Table S1: Top hits from target functional similarity classification of drug combinations at different thresholds for breast tissue

Supplementary Table S2: Drug synergy prediction for breast and colorectal cancer cell lines.

Supplementary Table S3: Comparison of synergy stratification workflow with supervised learning.

Supplementary Table S4: **Different settings for drug response prediction**

Supplementary Table S5: **Drug target information**
downloaded from https://www.cancerrxgene.org on March 2017.

Supplementary figures

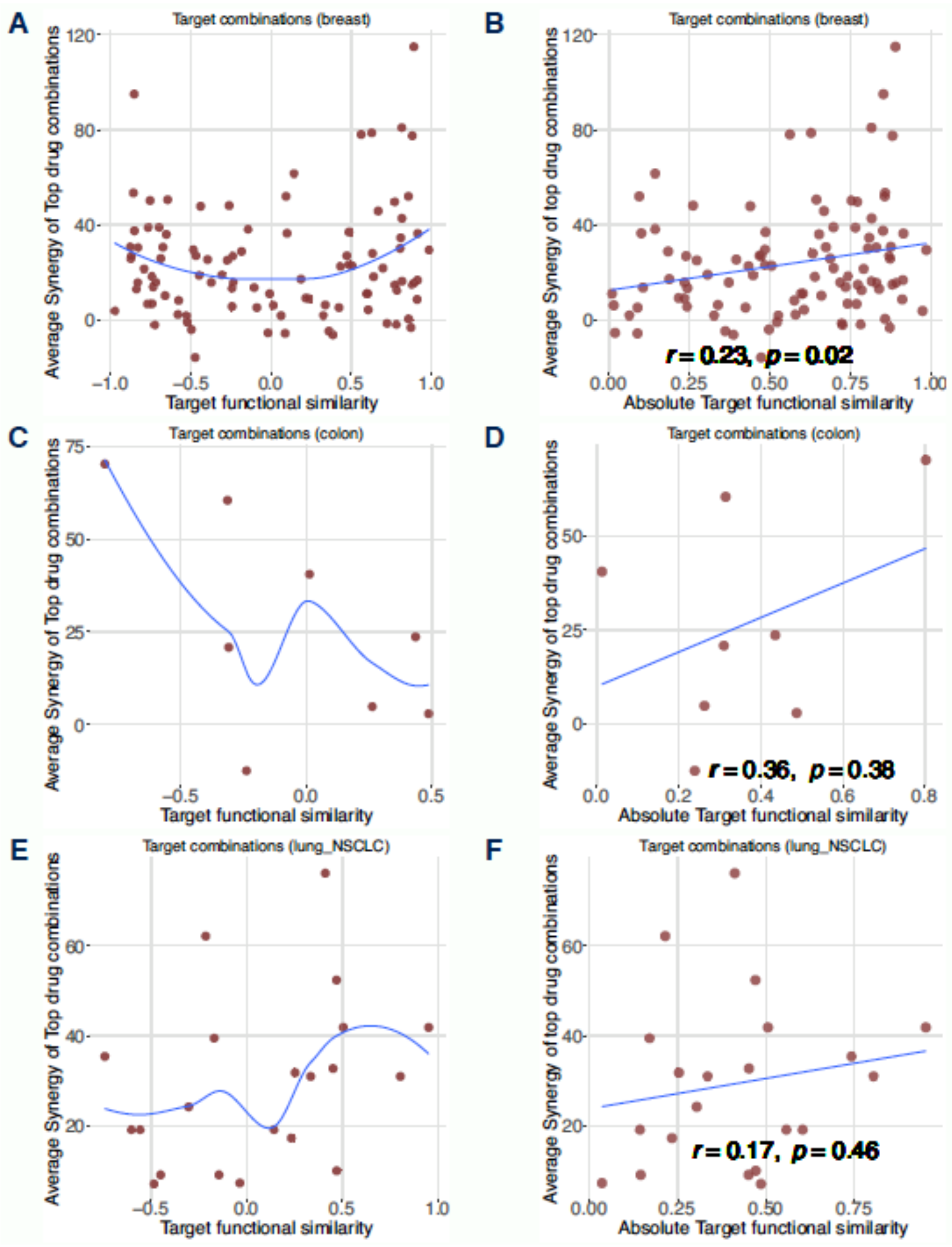

Supplementary Fig. S1: Influence of target functional similarity in drug synergy for AstraZeneca DREAM dataset.

The target functional similarity is the correlation between two target proteins by their interactions with the PROGENy pathways. For each tissue, we predicted the observed synergy using the target functional similarity and its absolute value. **A** and **B** for breast tissue. **C** and **D** for colon tissue. **E** and **F** for NSCLC lung tissue.

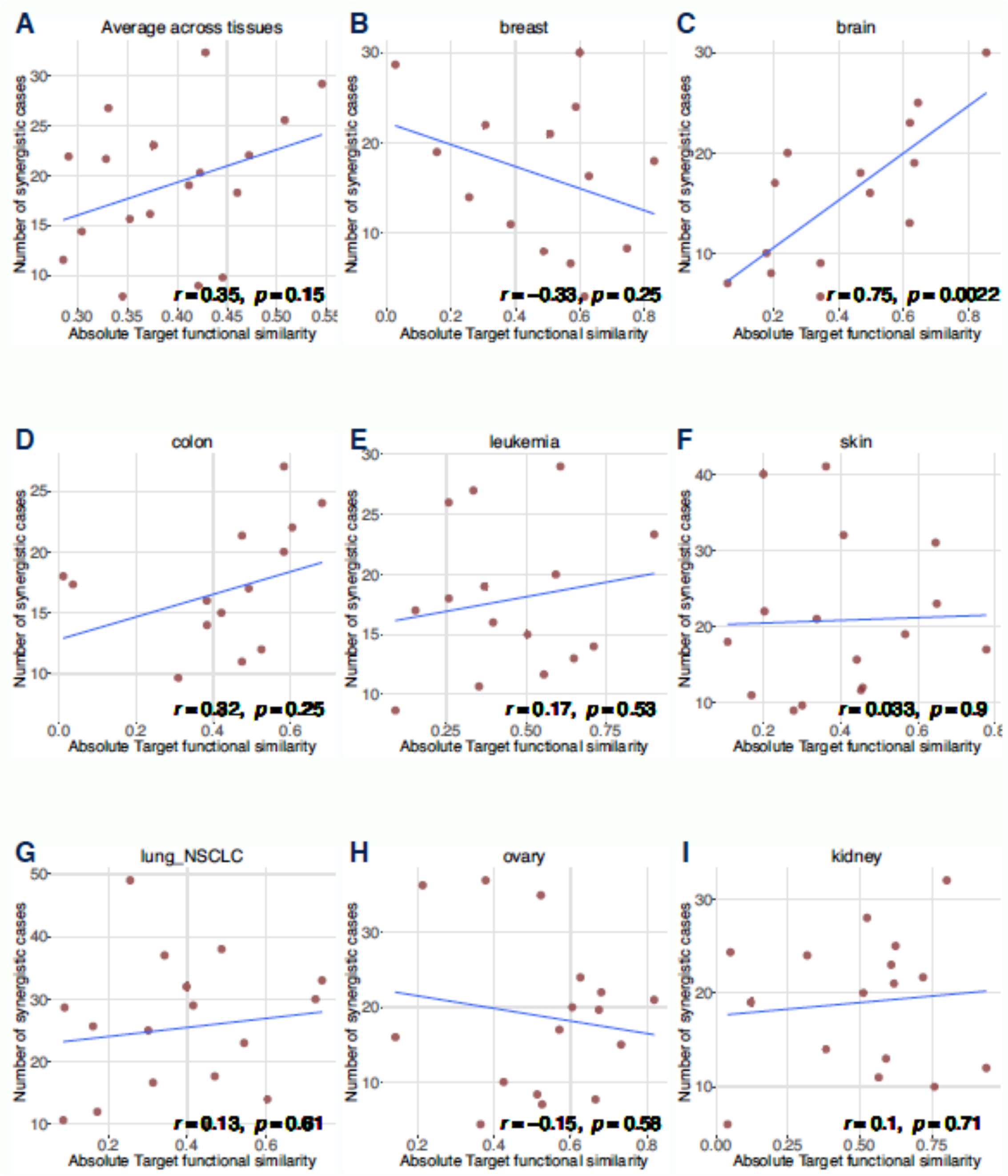

Supplementary Fig. S2: Influence of target functional similarity in drug synergy for NCI-ALMANAC dataset.

The target functional similarity is the correlation between two target proteins by their interactions with the PROGENy pathways. For each tissue, we predicted the number of synergistic combinations using the absolute target functional similarity.

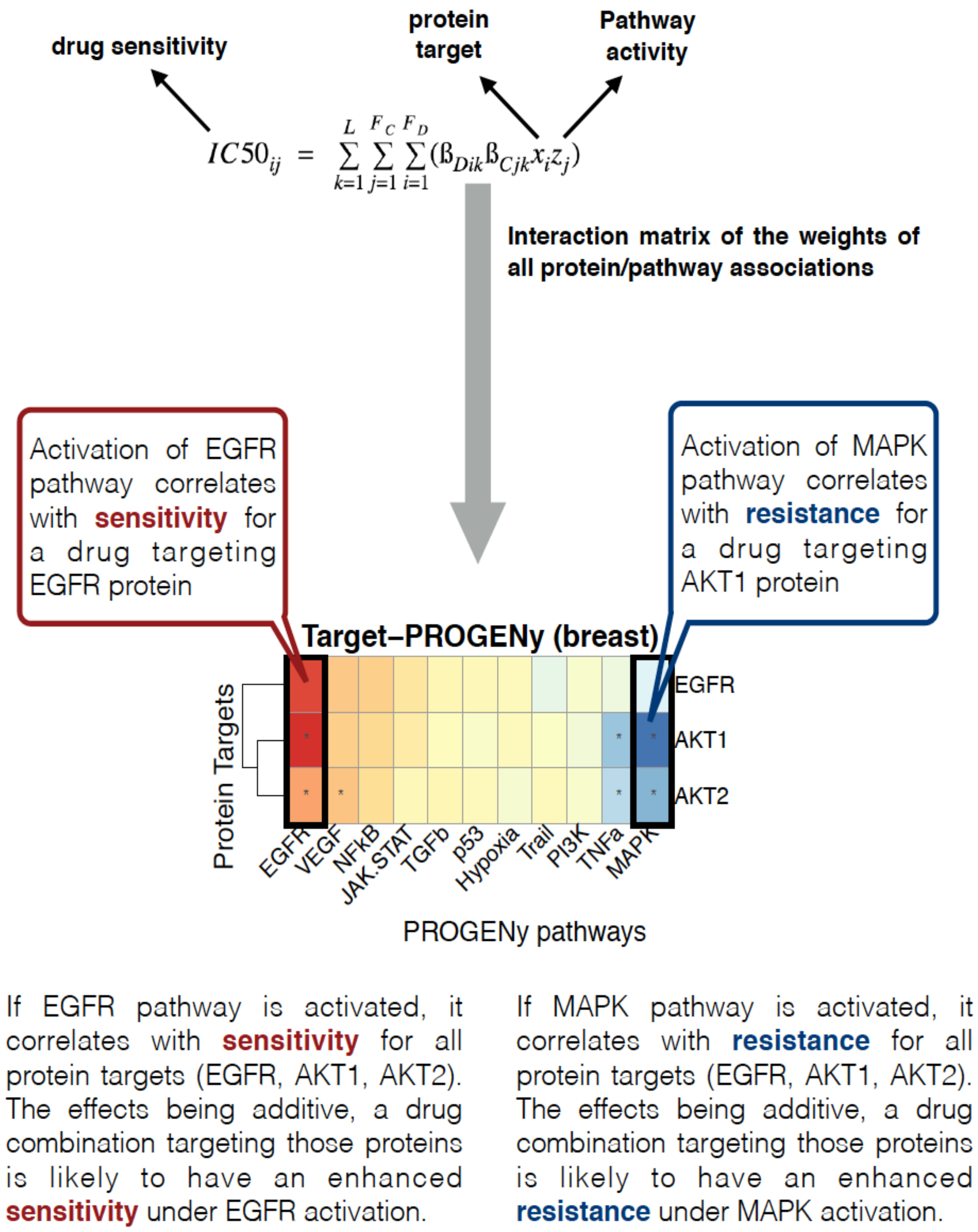

Supplementary Fig. S3: Interpretation of the interaction matrix.
Enhanced sensitivity occurs when targeting several proteins involved in drug response under the activation of the right pathway. The same rule applies to resistance.

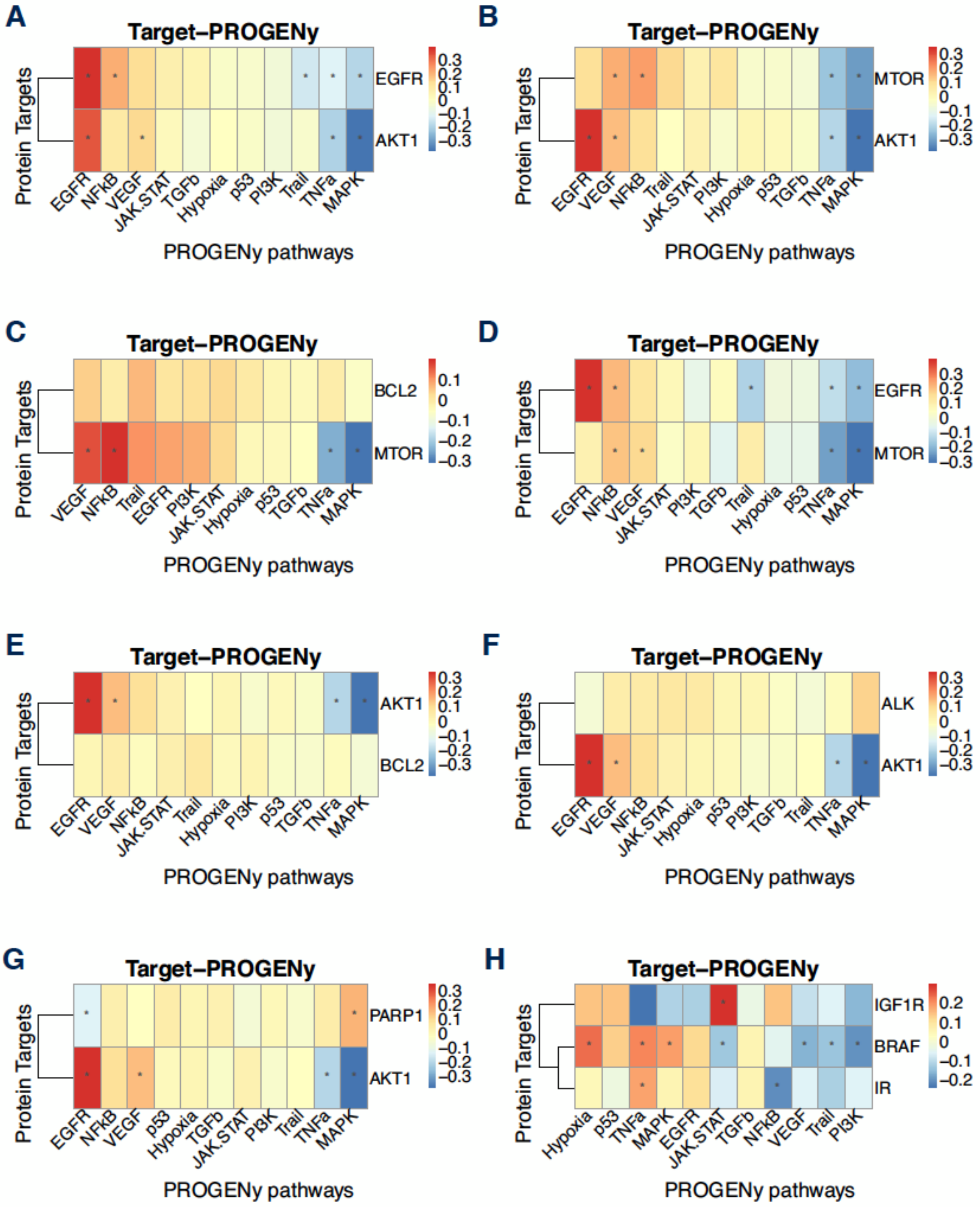

Supplementary Fig. S4: Functional profile of target proteins in breast and colorectal tissues. **A**, **B**, **C**, **D**, **E, F** and **G** describe the functional profile of AKT/EGFR, AKT/MTOR, BCL2/MTOR, EGFR/MTOR, AKT/BCL2, AKT/ALK and AKT/PARP1 pairs in breast tissue. **(h)** describes BRAF/IR's functional profile in colorectal tissue. The functional profile is a subset of the target pathway interaction in the Macau model. Pathways are ordered from the most sensitizing to the least. Significance of the interaction values is corrected as described in Yang *et al.*(Yang et al. 2018).

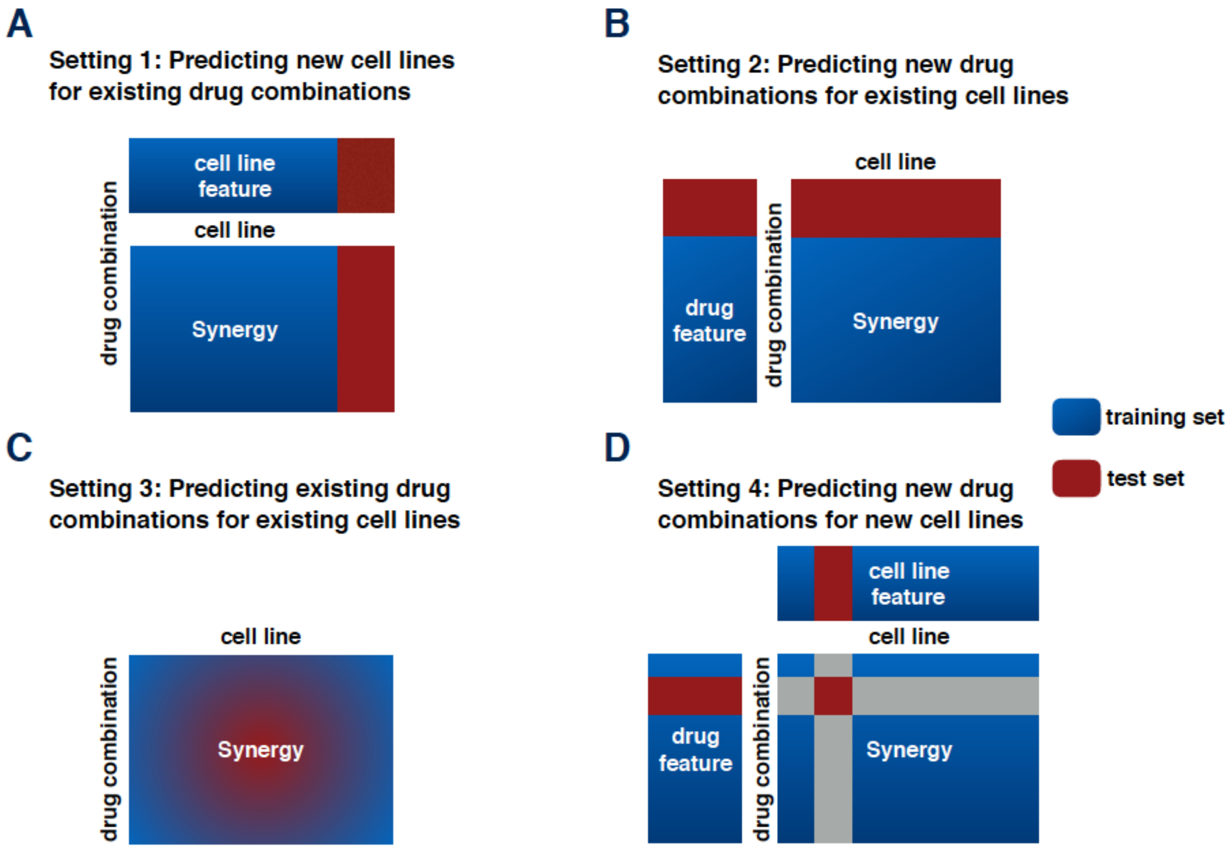

Supplementary Fig. S5: Different settings in drug synergy prediction.

A. Predicting new cell lines for existing drugs. For each drug pair, we compute the Pearson's correlation of observed versus predicted synergy across all cell lines of the test set.

B. Predicting new drug synergy for existing cell lines. For each cell line we compute the Pearson's correlation of observed versus predicted synergy across all drug pairs of the test set.

C. Predicting existing drug synergy for existing cell lines. This is a missing value imputation setting where side information of drug and cell lines are not required, but can be used to improve the result. The test data is defined by a percentage of the whole data set. We compute the Pearson's correlation of observed versus predicted synergy for all randomly chosen drugs - cell line triplets of the test set.

D. Predicting new drug synergy for new cell lines. We do two simultaneous cross validation on both drug and cell line sides. The test data is defined by association of the test set of the drug side with the test set of the cell lines side. We compute the Pearson's correlation of observed versus predicted synergy for all drug - cell line pairs of the test set.

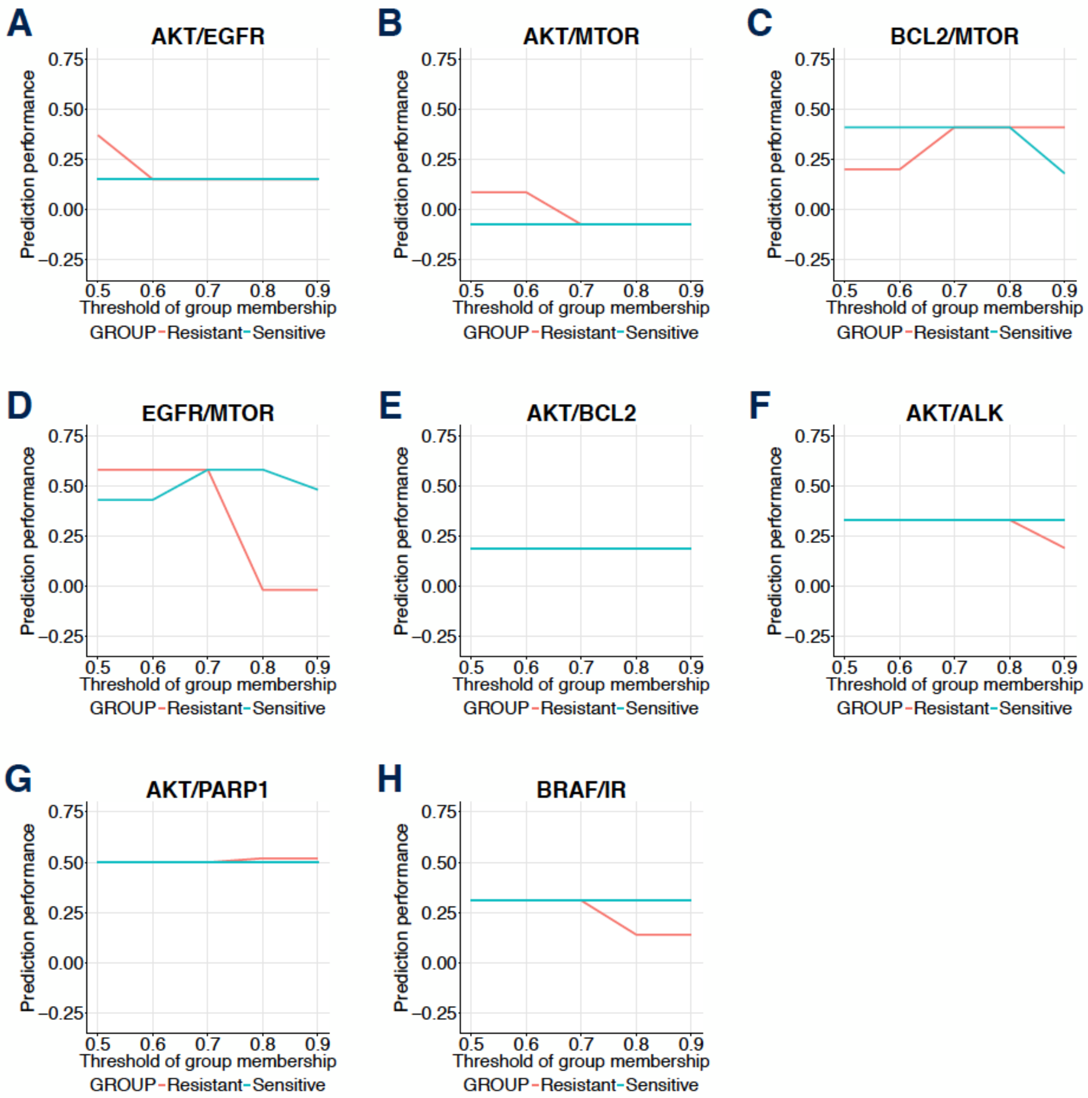

Supplementary Fig. S6: Sensitivity analysis for group membership parameters.
In the determination of Delta Pathway Equation, we explored the prediction performance for each target pair in AstraZeneca breast data and colorectal validation data, based on the following parameters: threshold for group membership of the top sensitive pathways and top resistant pathways.

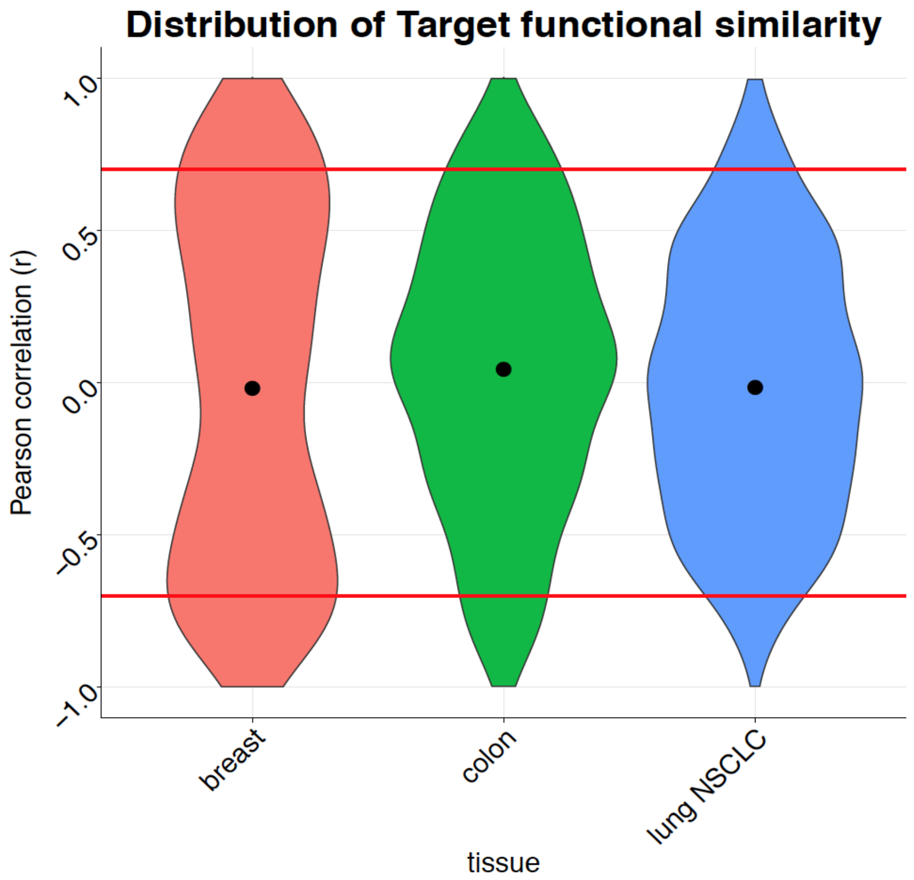

Supplementary Fig. S7: Distribution of similarity values across tissues.
For each tissue, we plotted the target functional similarities of the profiled target proteins and set the cut off of high similarity and high dissimilarity at 0.7 and -0.7.

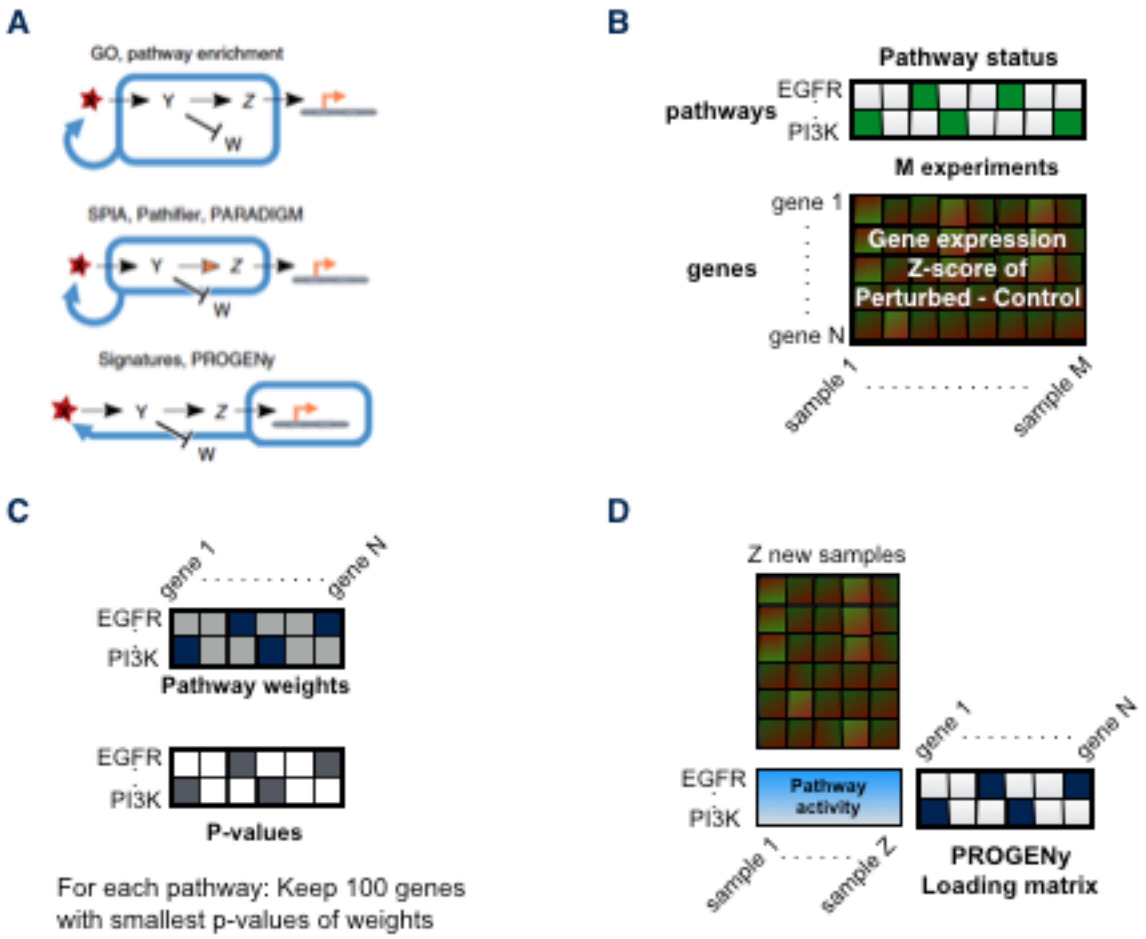

Supplementary Fig. S8: PROGENy pathway activities.
A. PROGENy is data driven pathway estimation method based on downstream perturbations. **B**. It uses gene expression z-score of perturbed - control from Array express perturbation experiments, and fits a linear model for each gene using pathway statuses (1/0/-1). **C**. We obtained a pathway weight matrix from the fitted model. And for each pathway we select the 100 smallest p-values and keep those genes while setting the other genes' weights to zeros. **D**. For new samples, we compute the pathway scores by multiplying the gene expression matrix with the pathway weight matrix.

Supplementary Table

Drug synergy prediction for breast and colorectal cancer cell lines

	Target pairs	Target functional similarity	Synergy model	Top sensitive pathways	Top resistant pathways	Delta Pathway Activity	Prediction performance from LOOCV
AstraZeneca breast data	AKT/EGFR (4 combinations)	0,9	Model 1	EGFR, NFkB, PI3K	MAPK	$\frac{EGFR+NFkB+PI3K}{3}-MAPK+CNV_{EGFR}$	0,15
	AKT/MTOR (5 combinations)	0,8	Model 1	EGFR, VEGF, PI3K	MAPK, TNFa	$\frac{EGFR+VEGF+PI3K}{3}-\frac{MAPK+TNFa}{2}$	0,086
	BCL2/MTOR (4 combinations)	0,7	Model 1	VEGF, NFkB, Trail	MAPK, TNFa	$\frac{VEGF+NFkB+Trail}{3}-\frac{MAPK+TNFa}{2}$	0,2
	EGFR/MTOR (6 combinations)	0,6	Model 1	EGFR, NFkB, VEGF	MAPK, TNFa	$\frac{EGFR+NFkB+VEGF}{3}-\frac{MAPK+TNFa}{2}+CNV_{EGFR}$	0,43
	AKT/BCL2 (4 combinations)	0,5	Model 1	EGFR, VEGF, PI3K	MAPK	$\frac{EGFR+VEGF+PI3K}{3}-MAPK$	0,19
	AKT/ALK (3 combinations)	-0,4	Model 2	EGFR, VEGF PI3K	MAPK, TNFa	$\frac{MAPK+TNFa}{2}-\frac{EGFR+VEGF+PI3K}{3}$	0,33
	AKT/PARP1 (3 combinations)	-0,8	Model 2	EGFR, VEGF PI3K	MAPK, TNFa	$\frac{MAPK+TNFa}{2}-\frac{EGFR+VEGF+PI3K}{3}$	0,50
Sanger colon data	BRAF/IR Dabrafenib/ BMS−754807	0,8	Model 1	Hypoxia, p53, MAPK	PI3K, VEGF, Trail	$\frac{Hypoxia+p53+MAPK}{3}-\frac{Trail+VEGF+PI3K}{3}$	All 48 cells: 0.31
						$\frac{Hypoxia+p53+MAPK}{3}-\frac{Trail+VEGF+PI3K}{3}+SNP_{BRAF}$	All 48 cells: 0.22<br>KRAS_mut: 0.5
						$\frac{Hypoxia+p53+MAPK}{3}-\frac{Trail+VEGF+PI3K}{3}+SNP_{KRAS}$	All 48 cells: 0.4

For 7 target pairs of the AstraZeneca breast cancer and 1 target pair of Sanger colorectal cancer data, we built prediction models based the general framework for predicting synergy (**Methods**).

Supplementary Table

Comparison of synergy stratification workflow with supervised learning

	Supervised learning	Hypothesis driven synergy stratification
Data source	Drug combination drug response on cancer cell lines	Single agent drug response on cancer cell lines
Input features	Gene expression and drug target	Gene expression and drug target
Additional information	mutation, CNV, cancer subtypes	mutation, CNV, cancer subtypes
Synergy prediction algorithm/model	Supervised learning algorithms such as tree based algorithms (Random Forest, XGBOOST) and matrix factorization.	Linear combination of gene expression derived pathway scores (from PROGENy)
Prediction settings (**Supplementary Figure 5**, **Supplementary Data 2**)	Setting 1: prediction of new cell lines for existing drugs<br>Setting 2: prediction of new drugs for existing cell lines<br>Setting 3: prediction of existing drugs for existing cell lines<br>Setting 4: prediction of new drugs on new cell lines	Setting 1: prediction of new cell lines for existing drugs<br>Setting 4: prediction of new drugs on new cell lines
Strength	(1) General purpose usage in drug wise and cell line wise settings<br>(2) Less dependant on domain expertise<br>(3) Easy to implement	(1) Does not require many drug combination experiments as prior knowledge<br>(2) Linear combination of pathway activation is suited for biological interpretation
Weakness	Requires an extensive set of drug combination drug response data	(1) Relies heavily on domain knowledge and literature evidence, making automated processing challenging<br>(2) Can only be used in a drug wise [illegible]
Translatability to *in vivo*	Requires the same data as the organism of interest for training.	Requires the tools to be built with the organism of interest.

We compare the pros and cons between predicting drug synergy using supervised machine leaning algorithms and our Hypothesis driven worflow.
